# Acute appendicitis in the context of De Garengeot hernia: a case report

**DOI:** 10.1093/jscr/rjac189

**Published:** 2022-07-23

**Authors:** Zachary Shellman, Dawit Worku, Nikhil Kulkarni

**Affiliations:** Department of General Surgery, Lincoln County Hospital, Lincoln, UK; Department of General Surgery, Lincoln County Hospital, Lincoln, UK; Department of General Surgery, Lincoln County Hospital, Lincoln, UK

## Abstract

De Garengeot hernia is a rare type of hernia so called when the vermiform appendix is found within the hernia sac of a femoral hernia. For the appendix to be inflamed is yet more uncommon. We present the case of a 61-year-old man who presented with a painful right groin lump. Computed tomography imaging reported an inguinal hernia containing a non-inflamed appendix however, intraoperative findings confirmed a femoral hernia containing an appendix with a necrotic tip. As such, these cases prove a diagnostic challenge as not only are the clinical findings illusive, but the radiological findings are often misleading. Diagnosis is often intraoperative and case reports such as this are useful in highlighting this challenging pathology.

## INTRODUCTION

De Garengeot hernia is an uncommon clinical entity resulting from the vermiform appendix entering a femoral hernia. In yet rarer situations the appendix can also be inflamed. Diagnosis can be challenging despite use of modern radiology techniques and often diagnosis is confirmed intraoperatively.

We present the case of a 61-year-old man who presented with a painful groin lump and was found to have De Garengeot hernia containing a necrotic appendix.

## CASE REPORT

A 61-year-old man who presented to the Emergency Department with a painful right groin lump. He was systemically well and afebrile.

By way of background, he was fit and well and did not suffer from any comorbidities. He took no regular medication and had no allergies.

On examination a tender groin lump was felt lateral to the pubic tubercle, inferior to the inguinal ligament. A femoral hernia was considered a more likely diagnosis.

Blood tests demonstrated a normal C-reactive protein and white cell count.

**Figure 1:**
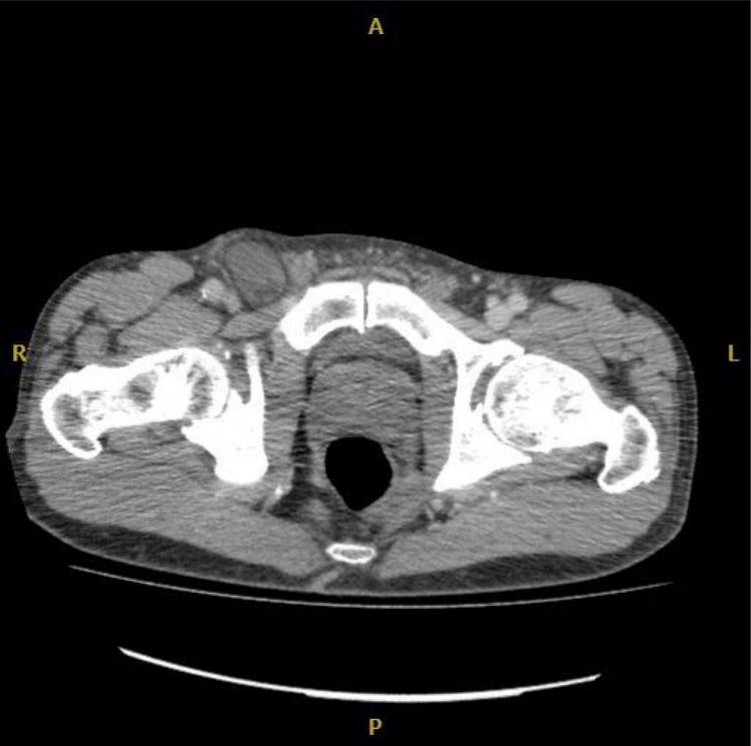
Axial CT image of right De Garengeot hernia.

**Figure 2:**
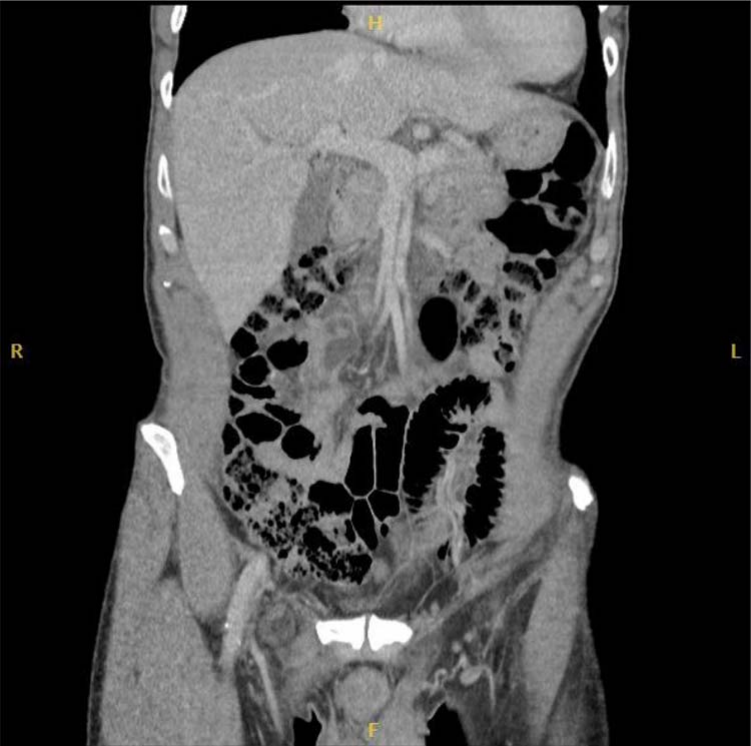
Coronal CT image of right De Garengeot hernia.

Computed tomography (CT) was reported as demonstrating an inguinal hernia containing fat and possibly the vermiform appendix (Figs 1 and 2). There was no conclusive evidence of appendicitis.

The patient was taken to theatre later that day for exploration via a modified McEvedy’s high approach. This demonstrated a hernia sac originating posterior to the inguinal ligament, emerging medial to the femoral vessels. A femoral hernia was diagnosed and exploration of the hernia sac demonstrating the vermiform appendix with necrosis of the distal segment. Appendicectomy was completed and primary suture repair used to repair the defect to the femoral canal.

The post-operative period was uneventful and the patient was discharged the following day.

## DISCUSSION

De Garengeot hernia is a rare subtype of femoral hernia and the presence of appendicitis is yet more uncommon. Femoral hernias account for 3–4% of all hernias [1]. These hernias are at higher risk of strangulation (~15–20%) due to the femoral canal’s rigid boarders and limited space [2]. De Garengeot hernia is thought to account for <1% of all femoral hernias. It is even rarer to find an inflamed appendix within the hernia sac (0.08–0.13%) [3].

Although it would be preferable not to use a mesh in a patient with well-documented inflammation, successful repair with mesh has been reported. Recent practices show a clear preference for primary suture hernia repair without mesh, with mesh repair in the presence of femoral hernia appendicitis only 21.6% [4]. Ultimately, the decision on whether to use a mesh or not depends on the surgeon’s preference in each individual case.

Our case highlights the shortcomings of CT imaging and the importance of clinical acumen in managing these patients. The intraoperative findings varied significantly from the more reassuring radiological findings. One possibility is the appendix at the time of the CT may not have been inflamed, suggesting these patients have a high propensity to develop appendicitis rapidly. In alternate, CT may not have been able to detect appendicitis within the hernia sac. Although CT has a sensitivity of 100% and a specificity of 98.9% in the diagnosis of routine abdominal acute appendicitis [5], the accuracy decreases significantly in femoral hernia appendicitis.

Reviews of De Garengeot hernia demonstrate radiological findings are not reliable in diagnosing this condition. In a 2013 review only 20% percent of ultrasound and 44% of CT studies were diagnostic, leading to an overall rate of 14% of femoral hernia appendicitis radiological diagnosis [3]. In another review from 2021 a pre-operative diagnosis of a De Garengeot hernia was established with imaging in only 31.5% of cases with CT found to have a sensitivity of 61.0% in diagnosing De Garengeot hernia [4]. This reinforces the importance of sound clinical judgment and suggests over reliance on imaging techniques is ill-advised in this context.

## CONFLICT OF INTEREST STATEMENT

None declared.
